# Skeletal Cell Fate Decisions Within Periosteum and Bone Marrow During Bone Regeneration

**DOI:** 10.1359/jbmr.081003

**Published:** 2008-10-13

**Authors:** Céline Colnot

**Affiliations:** Department of Orthopaedic Surgery, University of California at San Francisco, San Francisco General Hospital San Francisco, California, USA

**Keywords:** periosteum, bone marrow, bone graft, in vivo cell lineage, bone repair

## Abstract

Bone repair requires the mobilization of adult skeletal stem cells/progenitors to allow deposition of cartilage and bone at the injury site. These stem cells/progenitors are believed to come from multiple sources including the bone marrow and the periosteum. The goal of this study was to establish the cellular contributions of bone marrow and periosteum to bone healing in vivo and to assess the effect of the tissue environment on cell differentiation within bone marrow and periosteum. Results show that periosteal injuries heal by endochondral ossification, whereas bone marrow injuries heal by intramembranous ossification, indicating that distinct cellular responses occur within these tissues during repair. Next, lineage analyses were used to track the fate of cells derived from periosteum, bone marrow, and endosteum, a subcompartment of the bone marrow. Skeletal progenitor cells were found to be recruited locally and concurrently from periosteum and/or bone marrow/endosteum during bone repair. Periosteum and bone marrow/endosteum both gave rise to osteoblasts, whereas the periosteum was the major source of chondrocytes. Finally, results show that intrinsic and environmental signals modulate cell fate decisions within these tissues. In conclusion, this study sheds light into the origins of skeletal stem cells/progenitors during bone regeneration and indicates that periosteum, endosteum, and bone marrow contain pools of stem cells/progenitors with distinct osteogenic and chondrogenic potentials that vary with the tissue environment.

## INTRODUCTION

Bone regeneration is largely dependent on a successful inflammatory response, revascularization of the injury site, secretion of osteogenic and chondrogenic factors, and remodeling of the extracellular matrix within the damaged and new bone tissues.(1–6) Less is known about the origins of cells that produce bone and cartilage at the injury site. Several potential sources of skeletal stem cells/progenitors have been identified that may participate in bone repair. Cells may be delivered through the vasculature(7–9) and may be recruited from bone itself(10–14) or tissues immediately adjacent to bone, such as fat, tendon, and muscle.(15–17) Several lines of evidence suggest that the periosteum and the bone marrow are the main local sources of skeletal stem cells/progenitors for bone repair. Mechanical disruption of the periosteum or bone marrow delays healing,(18,19) presumably by removing the local source of cells. Although cells isolated from the periosteum or bone marrow can differentiate into chondrocytes and/or osteoblasts in vitro,(10,11,13,14) in vivo studies on the chondrogenic and osteogenic potentials of periosteum and bone marrow are limited.(20–22) Therefore, we still lack direct evidence showing the cellular contribution of periosteum and bone marrow to bone healing.

The difficulty in separating the role of various sources of cells during skeletal regeneration arises in part from the intricate structure of bone and the multiple tissues involved. In the majority of bone injuries, cortical bone is broken, thereby permitting communication between the periosteum, the bone marrow, and surrounding soft tissues. Moreover, when the physical barrier between bone compartments is disrupted, the healing response in one tissue may impinge on the response in the adjacent tissue through the diffusion of cells and growth factors. The goal of this study was to assess the extent to which periosteum and bone marrow contribute to osteogenic and chondrogenic lineages during bone repair and the extent to which the environment influences cell fate decisions in these tissues. In vivo cell lineage analyses were developed to track cells derived from periosteum, bone marrow, and endosteum, a specific compartment of the bone marrow lining the inner surface of bone. Bone grafts were collected from genetically labeled mice (Rosa26) and transplanted into wildtype hosts. Because the integrity of the periosteum, endosteum, and/or bone marrow was preserved during transplantation, labeled cells were recruited from their original niche and the fate of these cells was followed during bone healing. Results show that cells derived from the three tissues contribute differently to healing by intramembranous and endochondral ossification. This study also demonstrates that both intrinsic and environmental signals modulate cell fate decisions within periosteum, bone marrow, and endosteum during bone repair.

## MATERIALS AND METHODS

### Periosteal and endosteal/bone marrow injuries

All procedures followed protocols approved by the UCSF Animal Care and Use Committee. Adult C57B6 wildtype mice (males, 3–4 mo old) were anesthetized with an intraperitoneal injection of ketamine-metedomidin. To create periosteal injuries, an incision was made over the anterior-proximal tibia. The anterior-proximal tibial surface was exposed, and the periosteum was partially stripped using a razor blade. Tears covered an area of 3–4 mm in length. Wounds were closed with size 6-0 nylon sutures. After surgery, mice received subcutaneous injections of buprenorphine for analgesia and were allowed to ambulate freely. Mice were killed by cervical dislocation after anesthesia at days 3 (*n* = 3), 5 (*n* = 3), 7 (*n* = 6), 10 (*n* = 6), and 14 (*n* = 4) after surgery.

To create endosteal/bone marrow injuries, an incision was made at the knee joint. An insect pin (Fine Science Tools) was inserted from the joint into the intramedullary cavity, and the endosteum/bone marrow was reamed. Wounds were closed after removing the insect pin. Mice were killed at days 5 (*n* = 2), 7 (*n* = 6), 10 (*n* = 4), and 14 (*n* = 3).

### Preparation of bone grafts

Bone grafts were isolated from Rosa26 donors (males, 10 wk old) that express the *LacZ* reporter gene ubiquitously in the C57B6 background. Mice were killed after anesthesia. The tibias were collected free of skin and surrounding muscles. A fragment of cortical bone was cut (∼2 mm in length and 1mm in width) in the anterior-proximal area of each tibia using scissors. To follow cells derived from the periosteum, the endosteum and bone marrow were removed from the graft using a razor blade. The opposite was done to follow cells derived from endosteum and bone marrow. To follow cells derived from the endosteum, periosteum and bone marrow were removed but the endosteal surface was left intact. Negative controls were obtained by removing both the periosteum and bone marrow/endosteum from the graft, and positive controls by keeping intact both the periosteum and bone marrow/endosteum.

### Transplantation of bone grafts

Host C57B6 mice (males, 10 wk old) were prepared by creating a cortical defect on the anterior-proximal surface of the tibia. Under anesthesia, the tibial surface was exposed and a unicortical defect of ∼2 mm in length and 1 mm in width was created with a slow-speed dental engine using a 0.8-mm drill bit. To create nonstabilized fractures, three holes (0.4 mm in diameter) were drilled on the opposite side of the tibial cortex adjacent to the graft, and the bone was tapped until a fracture was created. Holes were not drilled in the opposite cortex of mice given grafts but no fracture.

The graft was placed in the cortical defect of host mice with or without fracture. The graft was oriented with the endosteum facing the marrow cavity of the host tibia or switched with the endosteum facing the soft tissue surrounding the external surface of the host bone. If needed, the size of the graft was adjusted to fit perfectly in the defect to prevent movement of the graft and to allow rapid integration within the cortex of the host tibia. The muscle was sutured over the defect to hold the graft in place, and wounds were closed. Mice were killed at days 5, 7, 10, and 14 (day 5, *n* = 2; day 7, *n* = 1 or 2 per group with normal orientation; days 10 and 14, *n* = 3–10 per group; Table 1).

**Table 1:** Proportions of Samples With Contribution of Donor PO, EO, and EO/BM to Bone and Cartilage During Bone Graft Healing and Nonstabilized Fracture Healing

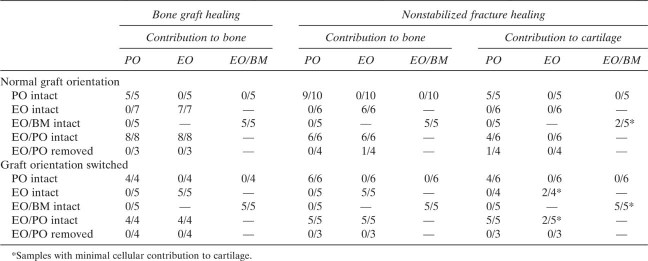

### Tissue processing and cell lineage analyses

Tibias were harvested and fixed for 24 h at 4°C in a solution containing 0.2% glutaraldehyde, 2 mM MgCl_2_, and 5 mM EDTA in PBS and washed three times for 1 h in a solution containing 2 mM MgCl_2_, 0.01% sodium-deoxycholate, and 0.02% NP40 in PBS. Samples were decalcified and cryo-embedded as described previously.(23) Longitudinal 8-μm-thick sections through the bone graft and the fracture callus were collected and stained with X-gal as previously described.(23) To identify which cell type the periosteum, bone marrow, and endosteum give rise to, Safranin-O/Fast Green and trichrome staining were performed to visualize cartilage and bone, respectively.

## RESULTS

### Comparing the mechanisms of healing in the periosteum and endosteum/bone marrow

**FIG. 1:**
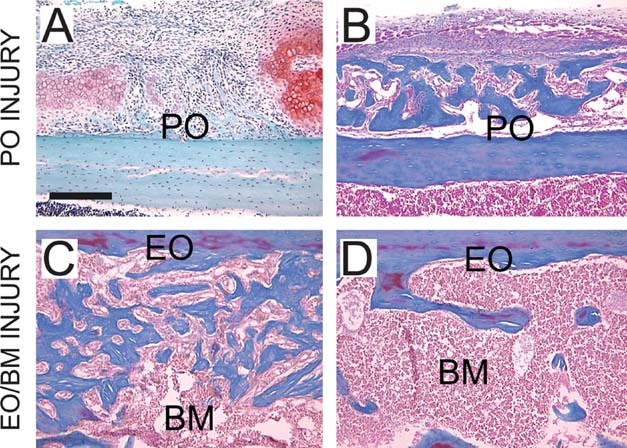
Periosteal injuries heal by endochondral ossification, whereas endosteal/bone marrow injuries heal by intramembranous ossification. Longitudinal sections through the mouse tibia stained with (A) Safranin-O/Fast Green (SO) and (B-D) trichrome (TC) at (A and C) 7 and (B and D) 14 days after (A and B) periosteal (PO) or (C and D) endosteal/bone marrow (EO/BM) injury. Scale bar = 200 μm.

To determine the intrinsic healing programs within periosteum and bone marrow/endosteum, injuries were created in one tissue without damaging the other. After periosteal injury, a thickening of the periosteum was observed at day 5 after surgery (data not shown). By day 7, cartilage and bone were detected along the periosteal surface (Fig. 1A). Cartilage was subsequently degraded and replaced by bone. By day 14, the periosteal surface was covered with woven bone that was being actively remodeled by osteoclasts (Fig. 1B and data not shown). These results indicate that injury to the periosteum induces repair through endochondral ossification. Conversely, reaming of the bone marrow/endosteal surface did not stimulate chondrogenesis. By day 7 after injury, the marrow cavity and endosteal surfaces were filled with new woven bone (Fig. 1C), which was largely resorbed by day 14 (Fig. 1D). Cartilage was not detected at any stages observed, showing that bone marrow/endosteal injuries healed by intramembranous ossification.

### Lineage analysis approach to follow periosteum- and bone marrow-derived cells during bone healing

**FIG. 2:**
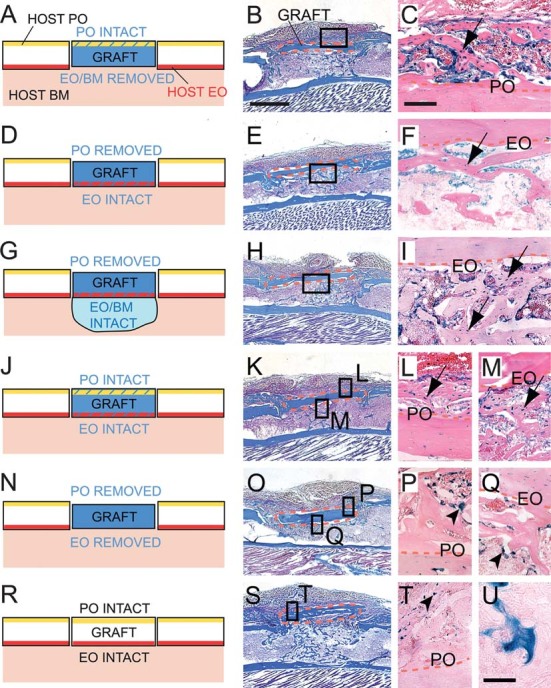
Periosteum and bone marrow/endosteum give rise to osteoblasts/osteocytes during bone graft healing. (Left) Schematic representations of Rosa 26 (blue) or wildtype bone grafts transplanted in the cortex of wildtype host tibias. Longitudinal sections through the mouse tibia stained with TC (middle) and adjacent sections stained with X-gal (right) at 14 days after bone grafting. X-gal-positive osteoblasts and osteocytes (arrow) are found at the intact periosteal surface (A-C), at the intact endosteal surface (D-F), within the marrow cavity (G-I), and at both periosteal and endosteal surfaces in positive controls (J-M), but not in negative controls (N-U). X-gal-positive osteoclasts express β-galactosidase ubiquitously (K, L, T, and U, arrowheads). Dotted orange lines delimit the bone graft. High magnifications correspond to boxed areas. Scale bars: B, E, H, K, O, and S = 1 mm; C, F, I, L, M, P, Q, and T = 100 μm; U = 20 μm.

To distinguish the role of the periosteum and bone marrow/endosteum in more complex healing environments, lineage analyses were performed using a combination of bone grafting and genetic labeling (Fig. 2). Bone grafts were harvested just before their transplantation, which allowed a high survival rate and successful integration of the grafts into the host bone. Graft survival was assessed based on the presence of X-gal-positive osteocytes throughout the cortex of the graft and X-gal-positive cells throughout the periosteum or bone marrow/endosteum at the surface of the graft. In the vast majority of samples (67 of 74 grafts total evaluated at days 10 and 14), osteocytes remained viable within the cortical bone grafts as shown by the maintained expression of β-gal throughout the course of healing. The graft did not survive in only seven samples, which were discarded from the study. In correlation with graft viability, the graft contributed to bone repair because X-gal-positive osteoblasts and osteocytes were present in the new bone being deposited.

### Tracking periosteum- and bone marrow-derived cells during bone graft healing

During healing of stabilized bone grafts, new bone formed at the periosteal and endosteal surfaces and within the marrow cavity (Fig. 2). Woven bone accumulating at the bone graft ends allowed bridging with the injured cortex. Cartilage was not detected at any stages including days 5, 7, and 10, indicating that bone grafts healed by intramembranous ossification. To assess the contribution of periosteum, endosteum, and bone marrow to bone graft healing, the distribution of graft-derived cells within the new bone was analyzed by X-gal staining on tissue sections. Grafts with periosteum intact gave rise to osteoblasts and osteocytes at the graft periosteal surface (Figs. 2A–2C; Table 1), whereas grafts with endosteum intact gave rise to osteoblasts and osteocytes at the endosteal surface (Figs. 2D–2F; Table 1). When the bone marrow was left attached to the endosteum, graft-derived osteoblasts and osteocytes were found in new bone both at the endosteal surface and within the marrow cavity (Figs. 2G–2I; Table 1). In positive controls, labeled cells were detected both at the periosteal and endosteal surfaces (Figs. 2J–2M; Table 1). In all these groups, graft-derived cells were also found at the junction of the graft and the old cortex, where a mixture of donor- and host-derived osteoblasts/osteocytes formed the new bone connecting the graft and the host cortex. In negative controls, no graft-derived osteoblasts and osteocytes were detected, showing that cells within the cortex of the graft did not participate in healing (Figs. 2N–2Q; Table 1). New bone was deposited by cells derived from the host and allowed bridging of the bone graft end; however, new bone did not span the entire periosteal surface as seen in samples with periosteum intact. X-gal-positive osteoclasts were present at the surface of new (Figs. 2P and 2Q, arrowheads) and old bone. Osteoclasts may have been derived both from the donor and/or the host, because these cells express β-galactosidase constitutively.(23–25)

### Tracking periosteum- and bone marrow-derived cells during nonstabilized fracture healing

To compare the contribution of periosteum, endosteum, and bone marrow to healing by endochondral ossification, a nonstable fracture was created adjacent to the graft at the time of bone grafting. Although fractures were not stabilized, bone healing was not delayed compared with stabilized fractures.(3,26) In this model, endochondral ossification is initiated at day 7.(26) The optimal time points to assess cartilage formation within the callus are days 10 and 14 postfracture, with a peak observed at day 10 and the beginning of cartilage replacement by bone at day 14.(3,26) Bone formation was also evaluated at these two time points.

**FIG. 3:**
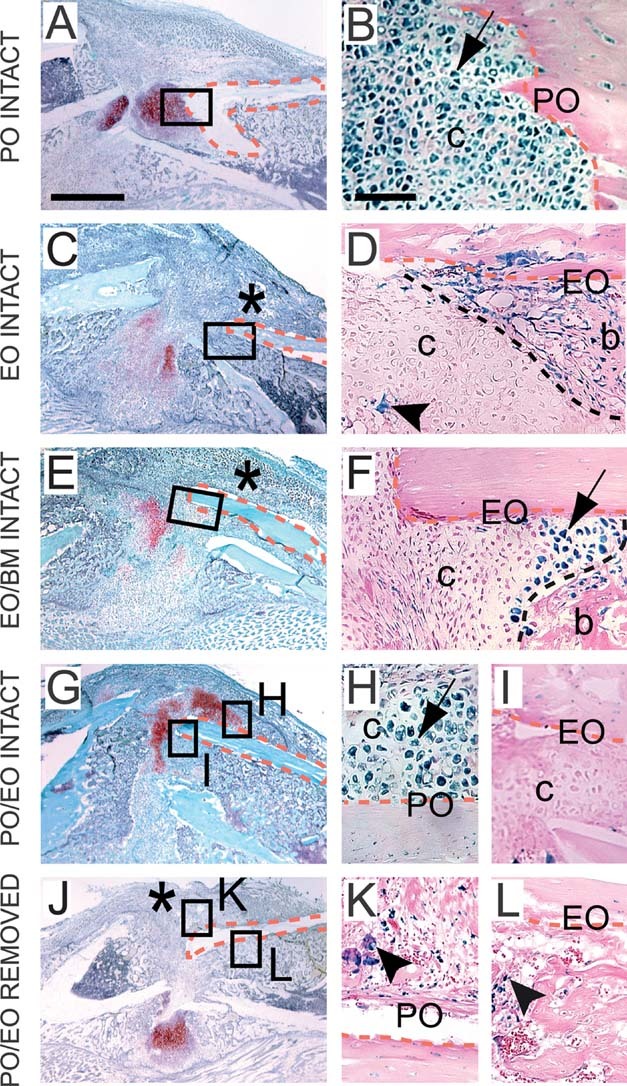
Chondrocytes are primarily derived from the periosteum during healing of nonstabilized fractures. Longitudinal sections through the fracture callus stained with SO (left) and adjacent sections stained with X-gal (right) 10 days after bone grafting and nonstable fracture. (A and B) Cartilage (c) forming at the intact periosteal surface stains positive with X-gal (arrow). Cartilage adjacent to the intact endosteal surface is (C and D) X-gal negative or (E and F) contains few X-gal-positive chondrocytes (arrow) when bone marrow is left intact. (G-I) In positive controls, X-gal-positive cartilage (arrow) is found at the periosteal surface and X-gal negative cartilage at the endosteal surface of the graft. (J-L) In negative controls, no X-gal-positive chondrocytes are detected. Endogenous β-galactosidase activity is detected in osteoclasts (arrowheads). Asterisk indicates absence of cartilage in the anterior part of the callus when the periosteum is removed. Dotted orange lines delimit the bone graft and black dotted lines delimit the junction between cartilage and bone within the callus. b, bone. Scale bars: A, C, E, G, and J = 1 mm; B, D, F, H, I, K, and L = 100 μm.

**FIG. 4:**
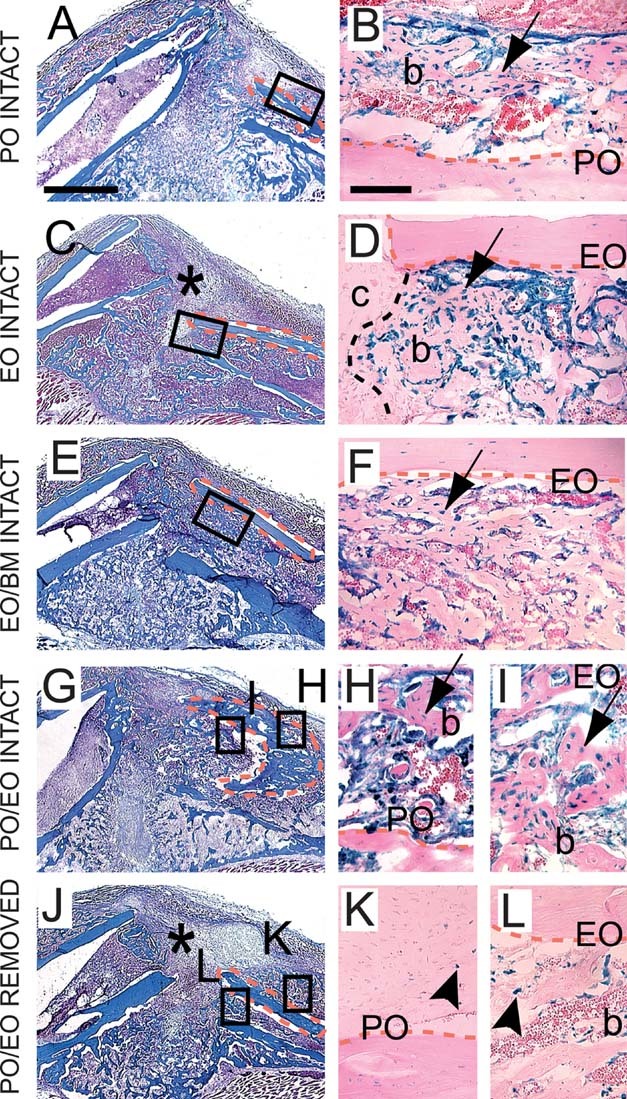
Osteoblasts/osteocytes originate from periosteum and endosteum/bone marrow during healing of nonstabilized fractures. Longitudinal sections through the fracture callus stained with TC (left) and adjacent sections stained with X-gal (right) 14 days after bone grafting and nonstable fracture. X-gal-positive osteoblasts and osteocytes (arrow) are found at the intact periosteal surface (A and B), at the intact endosteal surface (C and D), within the marrow cavity (E and F), and at both the periosteal and endosteal surfaces in positive controls (G-I), but not in negative controls (J-L). Osteoclasts exhibit endogenous β-galactosidase activity (K and L, arrowheads). Asterisk indicates decreased bone bridging in the anterior part of the callus when the periosteum is removed. Dotted orange lines delimit the bone graft and black dotted line delimits the junction between cartilage and bone. b, bone; c, cartilage. Scale bars: A, C, E, G, and J = 1 mm; B, D, F, H, I, K, and L = 100 μm.

As shown by histological analyses, the graft was integrated into the fracture callus (Figs. 3 and 4). In samples with periosteum intact, large cartilage islands that formed adjacent to the periosteal surface of the graft were always donor derived and therefore derived from the periosteum (Figs. 3A and 3B; Table 1). Cartilage that formed at a distance from the graft was host derived (data not shown). Endosteum-derived chondrocytes were not detected within the callus (Figs. 3C and 3D; Table 1). Endosteum/bone marrow-derived chondrocytes were detected in only two of five cases and constituted only a small proportion of the cartilage formed adjacent to the graft and close to the endosteal surface (Figs. 3E and 3F; Table 1). In positive controls, donor-derived chondrocytes were observed only at the periosteal surface of the graft (Figs. 3G–3I; Table 1). In negative controls, the graft did not give rise to cartilage excluding the possibility that chondrocytes may be derived from the cortex of the graft itself (Figs. 3J–3L; Table 1). One sample exhibited a small amount of cartilage at the periosteal surface, suggesting that the periosteum may not have been completely removed in this case (Table 1). As for all samples with periosteum removed, cartilage formation was reduced or inhibited at the proximity of the graft (Figs. 3C, 3E, and 3J, asterisks).

In parallel, we tracked graft-derived osteoblasts and osteocytes. Periosteum supplied osteoblasts and osteocytes at the periosteal surface (Figs. 4A, 4B, 4G, and 4H; Table 1). Endosteum and bone marrow provided osteoblasts and osteocytes at the endosteal surface and within the bone marrow (Figs. 4C–4G and 4I; Table 1). In negative controls, osteoblasts and osteocytes were host derived except in one case where endosteum was not completely removed (Figs. 4J–4L; Table 1). In the absence of periosteum, bone bridging was reduced at the periosteal surface (Figs. 4C, 4E, and 4J, asterisks). Removal of endosteum and bone marrow did not affect bone formation within the marrow cavity, indicating that the host endosteum/bone marrow may compensate for the absence of osteoblasts precursors attached to the graft.

### Effects of the tissue environment on cell differentiation within periosteum, bone marrow, and endosteum

**FIG. 5:**
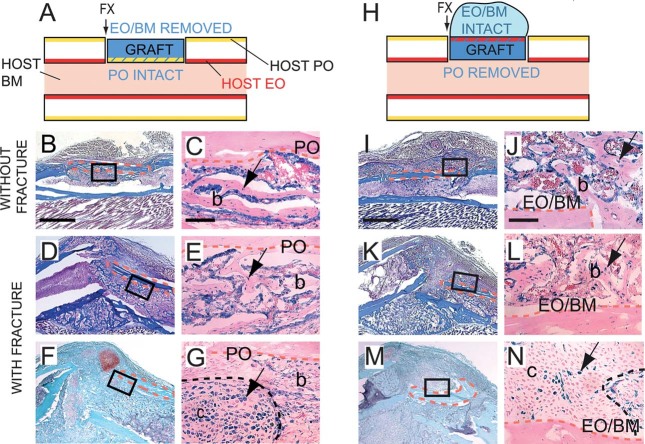
Effect of the tissue environment on cell differentiation in the periosteum and endosteum/bone marrow. (A and H) Schematic representation of Rosa 26 bone grafts (blue) with periosteum (PO, yellow) placed in the environment of endosteum/bone marrow or with endosteum/bone marrow (EO/BM, red) placed in the environment of the periosteum. Arrows indicates where the fracture is created. Sections through the bone graft were stained with TC (B, D, I, and K) and SO (F and M) and adjacent sections stained with X-gal (C, E, G, J, L, and N) at 10 (F, G, M, and N) and 14 (B-E and I-L) days after surgery. During bone graft (B and C) and nonstabilized fracture healing (D and E), X-gal-positive osteoblasts and osteocytes (arrow) derived from PO are found at the periosteal surface of the graft in the host bone marrow. (F and G) X-gal-positive chondrocytes (arrow) are detected in the same location. X-gal-positive osteoblasts and osteocytes (arrow) derived from EO/BM are located at the endosteal surface of the graft facing the host PO during bone graft (I and J) and nonstabilized fracture healing (K and L). (M and N) Only few X-gal-positive chondrocytes (arrow) are found in cartilage islands near the endosteal surface of graft. Dotted orange lines delimit the bone graft and black dotted line delimits the junction between cartilage and bone. b, bone; c, cartilage. Scale bars: B, D, F, I, K, and M = 1 mm; C, E, G, J, L, and N = 100 μm.

The differences among periosteum, endosteum, and bone marrow in their cellular contribution to bone repair may be caused by intrinsic differences within these tissues or extrinsic differences in their environment. To distinguish between intrinsic versus extrinsic effects, the fate of donor-derived cells was assessed when the orientation of the grafts were switched to place the periosteum in the environment of the endosteum/bone marrow and vice versa. The effects of the tissue environment on cell differentiation in the periosteum were assessed first (Fig. 5A). During healing of stabilized bone grafts, periosteum-derived cells differentiated into osteoblasts and osteocytes at the periosteal surface of the graft and within the marrow cavity of the host bone (Figs. 5B and 5C; Table 1). During healing of nonstabilized fractures, the periosteum gave rise to osteoblast/osteocytes (Figs. 5D and 5E; Table 1) and chondrocytes (Figs. 5F and 5G; Table 1) localized near the grafted periosteum and within the host bone marrow cavity. In all groups, no graft-derived cells were found at the periosteal surface of the host bone (data not shown). Next, the effects of the periosteal environment on cell differentiation in the endosteum and bone marrow were tested (Fig. 5H). Both endosteum and bone marrow gave rise to osteoblast/osteocytes at the endosteal surface of the graft during bone graft (Figs. 5I and 5J; Table 1) and nonstable fracture healing (Figs. 5K and 5L; Table 1). Cells derived from the donor endosteum or bone marrow were not detected within the marrow cavity of the host bone (data not shown). Within the fracture callus, donor endosteum and bone marrow generated few X-gal-positive chondrocytes in cartilage islands that were primarily host derived (Figs 5M and 5N; Table 1). The proportion of samples with endosteum- or endosteum/bone marrow-derived chondrocytes was increased compared with experiments with normal graft orientation (Table 1). Positive and negative controls were obtained as in previous experiments to confirm and validate the results (Table 1). Therefore, there were no changes in the osteogenic potentials of periosteum, endosteum, and bone marrow when the orientation of the grafts was reversed within the host bone. Likewise, placing the periosteum in the environment of the endosteum/bone marrow did not decrease its chondrogenic potential. In contrast, placing the endosteum and bone marrow in the environment of the periosteum increased their chondrogenic potential. Even in this ectopic environment, however, the endosteum/bone marrow was not as potent as the periosteum to support cartilage formation during fracture healing.

## DISCUSSION

### Cells are mostly recruited locally during bone repair

Whether bone healing is supported by multiple interchangeable sources of cells is still unclear. Lineage analyses show that the origin of osteoblasts and chondrocytes determines their location within the bone regenerate. Cells derived from the periosteum are always found at the periosteal surface, whereas cells derived from the endosteum and the bone marrow are always found at the endosteal surface or within the marrow cavity, respectively. Even when the tissues are transplanted ectopically, cells do not migrate from distant periosteal or bone marrow/endosteal sources within the callus because they are never detected at a distance from their original niche. Thus, bone itself seems to be the main local source of cells for bone repair.

Data provided here and previous studies further support this conclusion, because periosteum damage or removal affects osteogenesis and chondrogenesis and delays healing.(18,19) This delay most likely results from the removal of the local source of cells. Cells recruited from other sources might compensate for the lack of periosteum but take longer to arrive at the site of injury.(7,8),(15–17,27) Severe trauma and soft tissue damage have also been shown to impair bone healing,(28–30) which may be caused by the disruption of direct contacts between the periosteum and the bone matrix. These contacts may be important to preserve the stem cell niche and promote osteogenesis and chondrogenesis in vivo. Likewise, excessive mechanical disruption of the bone marrow delays fracture healing probably by deleting the mesenchymal stem cells (MSCs) and by disturbing the inflammatory response.(31,32)

### Distinct cellular contributions of periosteum, bone marrow, and endosteum to bone healing

Lineage analyses show that osteoblasts and osteocytes originate from periosteum, bone marrow, and endosteum, indicating that these three tissues contribute simultaneously to new bone forming by intramembranous and endochondral ossification. Chondrocytes within the fracture callus, however, are primarily derived from the periosteum. These results are consistent with the program of bone healing observed after periosteal or bone marrow/endosteal injuries, which heal by endochondral and intramembranous ossification, respectively. These data thus show that the periosteum supports both chondrogenesis and osteogenesis, whereas bone marrow/endosteum supports osteogenesis during bone repair.

Intrinsic differences between periosteum and bone marrow/endosteum influence cell fate decisions within these tissues. Whether periosteum is placed in its original environment or in the environment of the bone marrow, cells derived from the periosteum can give rise to osteoblasts/osteocytes or chondrocytes. Consequently, the lack of chondrocytes recruited from endosteum during nonstabilized fracture healing is not caused by the presence of inhibitors of chondrogenesis in the bone marrow but rather to distinct stem cell populations/progenitors. Hence, the osteogenic and chondrogenic potentials of the periosteum and bone marrow/endosteum may be defined by distinct stem cell populations/progenitors within these tissues. Intrinsic differences may also exist between endosteum and bone marrow because the capacity to undergo chondrogenesis was improved in samples with endosteum and bone marrow intact compared with endosteum alone. MSCs have been reported to reside in the stromal compartment of the bone marrow,(12,33,34) and results presented here now suggest that the endosteum may contain a distinct stem cell population within the bone marrow cavity. Stem cell populations/progenitors within periosteum and bone marrow/endosteum remain to be characterized at the cellular and molecular levels. Cells may respond differently to systemic growth factors by expressing various types of receptors or different levels of these receptors. Indeed, several reports indicate that cells within periosteum, endosteum, and bone marrow may not be equally sensitive to mechanical or biological stimuli.(35,36)

In addition, cell fate decisions within periosteum and bone marrow/endosteum are shown here to be regulated by the tissue environment. The degree of injury may influence the secretion of various growth factors that are distributed unevenly within the callus and elicit different cellular responses within periosteum, endosteum, and bone marrow. For example, prochondrogenic signals may be increased at the periosteal surface of bone. Moreover, previous work shows that the program of bone healing is largely influenced by the mechanical environment, which may impact cell differentiation.(3,4,26,37,38)

### Fate of cells in human therapies

A better understanding of the fate of cells within the fracture callus is necessary to assess the success of future cell-based therapies. Several methods can be used to enhance bone repair in human, such as iliac crest bone grafts, cortical bone grafts, and periosteal grafts.(39–44) Whether MSCs from bone marrow or other sources actually contribute to repair or support healing by providing factors inducing chondrogenesis or osteogenesis is not known.(45–48) MSCs from the bone marrow have been shown to differentiate into osteoblasts and chondrocytes in vitro and are largely used in multiple tissue engineering approaches.(49–51) Data show here that cells derived from bone marrow behave differently when they are recruited from their original niche because they mainly support osteogenesis in vivo. Although MSCs remain the most promising source of cells for human therapy, the mode of delivery and the fate of these cells once transplanted in vivo need to be considered. We previously showed that bone marrow transplantation was not a successful method to provide osteoblasts and chondrocytes to the fracture site.(4,23) We suspected that donor cells did not populate the MSC compartment of the host or that the engrafted MSCs were not recruited during healing. In addition, the contribution of bone marrow cells recruited from a distance or circulating stem cells/progenitors is still debatable.(9,52,53) Similarly, the contribution of surrounding soft tissues to bone healing is unclear.(54) Further study will be necessary to establish the relative contributions of various sources of stem cells/progenitors compared with the local periosteum and bone marrow/endosteum.

## Conclusion

Results from this study provide direct evidence that the periosteum, endosteum, and bone marrow are major sources of skeletal stem cells/progenitors and contribute differently to osteogenesis and chondrogenesis during bone repair. The distinct cellular contributions of periosteum, endosteum, and bone marrow suggest both intrinsic differences within the populations of stem cells/progenitors residing in these tissues and differences in their tissue environment. Establishing the nature of these differences and, in particular, identifying the sources of adult skeletal stem cells/progenitors in vivo, will have profound implications for the treatment of recalcitrant fractures and bone diseases.
